# Preliminary Evaluation of the Safety and Probiotic Potential of *Akkermansia muciniphila* DSM 22959 in Comparison with *Lactobacillus rhamnosus* GG

**DOI:** 10.3390/microorganisms8020189

**Published:** 2020-01-30

**Authors:** Autilia Cozzolino, Franca Vergalito, Patrizio Tremonte, Massimo Iorizzo, Silvia J. Lombardi, Elena Sorrentino, Delia Luongo, Raffaele Coppola, Roberto Di Marco, Mariantonietta Succi

**Affiliations:** 1 Department of Agricultural, Environmental and Food Sciences (DiAAA), University of Molise, Via De Sanctis, 86100 Campobasso, Italy; a.cozzolino@studenti.unimol.it (A.C.); franca.vergalito@unimol.it (F.V.); tremonte@unimol.it (P.T.); iorizzo@unimol.it (M.I.); silvia.lombardi@unimol.it (S.J.L.); coppola@unimol.it (R.C.); succi@unimol.it (M.S.); 2 Institute of Biostructure and Bioimaging of the National Research Council (IBB-CNR), Via Mezzocannone 16, 80134 Napoli, Italy; delia.luongo@unina.it; 3 Department of Medicine and Health Sciences “Vincenzo Tiberio”, University of Molise, Via De Sanctis, 86100 Campobasso, Italy; roberto.dimarco@unimol.it

**Keywords:** *Akkermansia muciniphila*, *Lactobacillus rhamnosus* GG, hydrophobicity, auto-aggregation, co-aggregation, biofilm formation, co-culture

## Abstract

In this study, for the first time, we examined some of the physico-chemical properties of the cell surface of *Akkermansia muciniphila* DSM 22959, comparing it with those of *Lactobacillus rhamnosus* GG—one of the most extensively studied probiotic microorganisms. In particular, hydrophobicity, auto-aggregation, co-aggregation, and biofilm formation were investigated. In addition, antibiotic susceptibility, co-culture, and antimicrobial activity of the two strains were compared. Hydrophobicity was evaluated using xylene and toluene, showing that *A. muciniphila* DSM 22959 possessed moderate hydrophobicity. *A. muciniphila* showed a faster and higher auto-aggregation ability than *Lb. rhamnosus* GG, but a lower aptitude in biofilm formation. In the co-aggregation test, the best performance was obtained by *Lb. rhamnosus* GG. Regarding the susceptibility to antibiotics, the differences between the two strains were remarkable, with *A. muciniphila* DSM 22959 showing resistance to half of the antibiotic tested. Interesting results were also obtained with regard to the stimulating effect of *Lb. rhamnosus* GG on the growth of *A. muciniphila* when co-cultured.

## 1. Introduction

*Akkermansia muciniphila* is an oval shaped, gram-negative, strictly anaerobic bacterium belonging to the *phylum* Verrucomicrobia, and it constitutes 3% to 5% of the gut microbial community in both human and other mammalian species [1,2]. 

It was firstly isolated from a human fecal sample in 2004 [3], and interest in this microorganism increased during time due to its ability to release enzymes into the intestinal tract that help to regulate the balance of mucin, a major component of the mucous layer that resides on the surface of the gastrointestinal mucosa [4]. Human mucin degradation by *A. muciniphila* provides competitive exclusion against pathogenic mucus degraders and may have a gatekeeping and signaling function [5]. To gain a competitive advantage, *A. muciniphila* has evolved to metabolize the complex glycans provided by the mucus layer as its sole carbon and nitrogen source [6,7]. An efficient colonization of *A. muciniphila* was observed with the highest numbers in the cecum, where most of the mucin is produced [8]. Mucus degradation leads to the production of metabolites, such as acetic acid, propionic acid and oligosaccharides, which may play a role in metabolic health or inflammatory host status [9]. 

Some authors observed that *A. muciniphila* is abundant in biopsies of healthy subjects and reduced in those of patients with inflammatory bowel disease, obesity, and other diseases [8]. Therefore, due to its low presence in people affected by intestinal disorders and its link with the wellbeing of the mucus layer, *A. muciniphila* was suggested as a biomarker for the intestinal health [9].

On the other hand, Weir et al. [10] observed that mucin-degrading bacteria, including *A. muciniphila*, were present in a significantly larger proportion in the feces of colon cancer patients than in healthy subjects. However, they hypothesized that the observed increase in *A. muciniphila* may be due to an increase of substrate availability as some types of mucin were overexpressed in colon cancers. 

Several authors stated that *A. muciniphila* exerts a range of biological activities positively related to human health; it has therefore been widely considered as a novel potential candidate to ameliorate metabolic disorders associated with obesity, diabetes, liver diseases, and cardiometabolic disorders [11]. Indeed, its administration has been shown to profoundly reduce the development of such diseases [12,13]. 

In addition, this microorganism has been identified in human milk samples immediately after delivery (colostrum), as well as after 1 and 6 months of breastfeeding [14]. Currently, the species is not included by EFSA (European Food Safety Authority) in the QPS (Qualified Presumption of Safety) list [15]. However, all the knowledge acquired so far on this species supports the proposal of *A. muciniphila* as a next generation probiotic [12].

According to the FAO (Food and Agriculture Organization) and WHO (World Health Organization) [16] recommendations, probiotic strains must meet specific criteria of safety and functionality. For these reasons, this study has been undertaken to preliminary evaluate some features, such as antibiotic susceptibility, physico-chemical properties of bacterial cell surface, biofilm formation and antimicrobial activities, of the human isolated strain *A. muciniphila* DSM 22959. Studied properties were compared with those of *Lactobacillus rhamnosus* GG, one of the most widely and extensively studied probiotic strain [17,18] belonging to a species that is generally recognized as safe (GRAS) [19]. 

## 2. Materials and Methods

### 2.1. Bacterial Strains

The bacterial strains used in this study were *Lactobacillus rhamnosus* GG, previously isolated from a pharmaceutical preparation (Valio LTD, Helsinki, Finland) as described by Succi et al. [20]; *Akkermansia muciniphila* DSM 22959; *Escherichia coli* DSM 5698, and *E. coli* K12-DH5 (DSMZ, Braunschweig, Germany); *Proteus mirabilis* ATCC 29906; *Enterococcus faecalis* ATCC 2912; *Staphylococcus aureus* ATCC 29213; *Lactobacillus acidophilus* ATCC 4356 (ATCC—American Type Culture Collection). All the strains were maintained at −80 °C in glycerol [21] and propagated twice at 37 °C in proper media before their use.

### 2.2. Antibiotic Susceptibility Test

The antibiotic susceptibility of *Lb. rhamnosus* GG and *A. muciniphila* DSM 22959 was performed with the Etest (Epsilometer test) gradient technology (Biomerieux, Marcy-l’Etoile, France). The antibiotics were selected on the basis of the EFSA document regarding bacteria of human importance and the cut-off values are those indicated in the same document [22].

The Etest strips of chloramphenicol, clindamycin, ampicillin, gentamicin, tetracycline, streptomycin, kanamycin, and erythromycin were used in the concentration range 0.016–256 µg/mL. BHI agar plates (Oxoid Ltd., Hampshire, UK) for *A. muciniphila* DSM 22959 and LSM agar plates (90% of iso-sensitest—IST, and 10% of MRS, Oxoid) for *Lb. rhamnosus* [23] were inoculated with bacterial suspensions in a sterile saline solution. 

The bacterial cell density of suspensions was adjusted to match McFarland turbidity standard 0.5 using a spectrophotometer (bio-spectrometer basic, Eppendorf, Italy). After drying the surfaces of the plates for 15 to 20 min, Etest strips of tested antibiotics were applied directly onto the surface. The plates were incubated overnight under anaerobic conditions at 37 °C. After the required incubation period, the MIC values were read, detecting the pointed end of the inhibition ellipse that intersects the side of the strip.

### 2.3. Hydrophobicity Assay 

The determination of cell surface hydrophobicity was evaluated on *Lb. rhamnosus* GG and *A. muciniphila* DSM 22959 based on the bacterial ability to adhere to hydrocarbons. Bacterial adhesion to hydrocarbons (BATH) test was performed using xylene and toluene, according to the procedure described by Collado et al. [24] with some modifications. Briefly, *Lb. rhamnosus* GG and *A. muciniphila* DSM 22959 were grown overnight at 37 °C in MRS broth and BHI broth, respectively. Cells were collected by centrifugation (8000 rpm for 10 min at 4 °C), washed twice and re-suspended in a sterile phosphate buffer saline (PBS, pH 7) to achieve an optical density (OD 580 nm) of 0.5 (±0.05), in order to standardize the number of bacteria (7–8 Log CFU/mL). Then, an equal volume of hydrocarbon (xylene or toluene) was added. The two-phase system was thoroughly mixed by vortexing for 5 min. The aqueous phase was carefully removed after 15, 30, and 60 min of incubation at room temperature and its absorbance at 580 nm was measured using a spectrophotometer. Affinity to hydrocarbons (hydrophobicity) was calculated using the following formula:

(1) $$\mathrm{Hydrophobicity}\;\%=(\frac{OD_{0}-OD_{final}}{OD_{0}})\times 100,$$

where OD_0_ and OD_final_ are the absorbance values before and after extraction with hydrocarbons, respectively.

Hydrophobicity was calculated as the percentage decrease in the optical density of the initial bacterial suspension, as the hydrophilic bacteria will be located in the water, while the hydrophobic bacteria will be associated with hydrocarbons [24].

### 2.4. Auto-Aggregation Assay 

The auto-aggregation assay was performed as described by Collado et al. [24]. Briefly, cell suspensions of *Lb. rhamnosus* GG and *A. muciniphila* DSM 22959 were prepared as described above and incubated at 37 °C. OD was adjusted to 0.5 (±0.05). Then the bacterial suspensions were incubated at 37 °C and their OD were detected at 1, 2, 5, and 24 h. 

Auto-aggregation percentage was calculated using the following formula [25]:

(2) $$\mathrm{Auto}-\mathrm{aggregation}\;\%=1-(\frac{OD_{final}}{OD_{0}})\times 100,$$

where OD_0_ is the absorbance at time 0, and OD_final_ is the absorbance detected after 1, 2, 5, and 24 h.

### 2.5. Biofilm Formation

Biofilm formation was evaluated on *Lb. rhamnosus* GG and *A. muciniphila* DSM 22959 under static conditions, as described by Stepanović et al. [26] with some modifications. Strains were grown overnight at 37 °C in tryptic soy broth (TSB, Oxoid). Then, bacterial cells were harvested by centrifugation at 8000 rpm for 10 min at 4 °C, washed twice with PBS and resuspended in the following media: TSB without glucose and TSB supplemented with 0.25%, 1%, and 2.5% D-glucose. Three aliquots of 200 µL of each bacterial suspension were filled in a 96-well polystyrene microtiter plate. Negative controls were constituted by wells filled with uninoculated culture media. Microtiter plates were incubated for 24 h at 37 °C. The medium was removed by each well and plates were washed three times with a sterile saline solution to remove unattached cells. The remaining attached cells were fixed with 200 µL of 99% methanol (Sigma–Aldrich) per well. After 15 min, wells were emptied and left to dry. Then wells were stained for 5 min with 200 μL of 2% Crystal Violet (Liofilchem, Italy) per well. Excess stain was removed by washing three times with sterile saline solution. After the plates were air-dried, the adherent cells were resuspended in 160 µL of 33% (v/v) glacial acetic acid (Sigma–Aldrich). The OD of each well was measured at 580 nm by using an automated PerkinElmer 1420 Multilabel Counter.

The cut-off (OD_C_) was defined as the mean OD value of the negative control. Based on the OD, strains were classified as non- biofilm producers (OD ≤ OD_C_), weak (OD_C_ ˂ OD ≤ 2 × OD_C_), moderate (2 × OD_C_ ˂ OD ≤ 4 × OD_C_), or strong biofilm producers (4 × OD_C_ ˂ OD).

### 2.6. Co-Culture of Lb. rhamnosus and A. muciniphila

This test was performed following the protocol described by Ruiz et al. [27] with some modifications. Strains were grown at 37 °C in BHI broth, cells were collected by centrifugation (8000 rpm for 10 min at 4 °C) from the early logarithmic growth phase, washed twice, and re-suspended in sterile saline solution to an optical density (580 nm) of about 0.5. The cell suspensions were individually (only *Lb. rhamnosus* GG and only *A.* *muciniphila* DSM 22959) inoculated (1% v/v) in tubes of BHI broth preheated at 37 °C, and incubated at 37 °C. The same procedure was used on the strains in co-culture. In this last case, the co-culture tubes were vigorously vortexed before incubation in order to promote the mix between the two bacteria. At time 0, and after 3, 6, and 24 h of incubation, microbial counts were performed on single cultures *Lb. rhamnosus* GG and *A. muciniphila* DSM 22959, and on the co-culture. For co-culture, *Lb. rhamnosus* GG was counted in MRS agar added with kanamycin at 37 °C in anaerobiosis. *A. muciniphila* DSM 22959 was counted on BHI agar added with chloramphenicol at 37 °C in anaerobiosis.

### 2.7. Co-aggregation Assay

The ability of *Lb. rhamnosus* GG and *A. muciniphila* DSM 22959 to co-aggregate with *E. coli* DSM 5698, *E. coli* K12-DH5, *P. mirabilis* ATCC 29906, *Ec. faecalis* ATCC 2912, *S. aureus* ATCC 29213, and *Lb. acidophilus* ATCC 4356 was evaluated following the procedure reported by Collado et al. [24]. 

Bacterial suspensions were prepared as described for BATH test. Equal volumes (500 μL) of *Lb. rhamnosus* GG or *A. muciniphila* DSM 22959 were mixed with each of the other strains for 10 s on a vortex and incubated at 37 °C without agitation.

Absorbance (580 nm) of each mix, and of each bacterial suspension (controls) was monitored at 5 and 24 h of incubation. The percentage of co-aggregation was calculated using the following formula [28]:

(3) $$\mathrm{Co}-\mathrm{aggregation}\;\%=(\frac{[(OD_{x}+OD_{y})/2]-OD_{mix}}{(OD_{x}+OD_{y})/2})\times 100,$$

where OD_x_ and OD_y_ represent the absorbance values of the separate bacterial suspensions in control tubes, and OD_mix_ represent the absorbance values of the mixed bacterial suspensions at 5 and 24 h. 

### 2.8. Antimicrobial Activity 

The antimicrobial activity of *Lb. rhamnosus* GG and *A. muciniphila* DSM 22959 (producers) was evaluated against *E. coli* DSM 5698, *E. coli* K12-DH5, *P. mirabilis* ATCC 29906, *Ec. faecalis* ATCC 2912, *S. aureus* ATCC 29213 and *Lb. acidophilus* ATCC 4356 (indicators).

The antimicrobial activity of the strains was conducted according to the agar well diffusion assay described by Tremonte et al. [29] with same modifications. Briefly, 20 mL of BHI soft agar (0.7% agar) inoculated with an overnight culture of each indicator strain (final concentration of about 7 Log CFU/mL) were poured in Petri plates. Wells of 5.0 mm in diameter were bored into plates and 50 μL of each producer strain was placed into each well. After incubation at 37 °C for 24 to 48 h, the plates were observed for the inhibition zones and the inhibition halos were normalized using the following formula [30]:

Inhibition score (IS) = Ø inhibition halo (mm)/Ø well (mm).(4)

On this basis, the antimicrobial effect was considered as low (1 < IS <3), moderate (3 ≤ IS < 5), strong (5 ≤ IS < 7) or very strong (7 ≤ IS < 9).

A calibrated-densitometer (GS-800, Bio-Rad, Hermles CA, USA) was used for imaging acquisition and Adobe Photoshop CS4 Extended software was used for the measurement of clearing zones. 

### 2.9. Statistical Analysis

All data were expressed as mean ± standard deviation (SD) of the measurements were carried out in duplicate on three independent experiments. Statistical analysis was performed through the analysis of variance (ANOVA) followed by the Tuckey’s multiple comparison. Statistical significance was attributed to *p* values < 0.05. The software SPSS (IBM SPSS Statistics 21) was used for the analysis.

## 3. Results and Discussion

This study aimed at the preliminary evaluation of some probiotic features of the human isolated strain *A. muciniphila* DSM 22959. The strain *Lb. rhamnosus* GG was chosen for comparative purposes, considering that it is one of the most extensively studied and well characterized probiotic strains [31,32,33,34]. In addition, *Lb. rhamnosus* GG is widely consumed worldwide and it is also used in many pharmaceutical formulas and fermented milk-based preparations [17,18].

### 3.1. Antimicrobial Susceptibility

Antibiotic response is a very important feature for selecting probiotic strains. In fact, probiotics should have low antibiotic resistance and should not possess transmissible antibiotic resistance that can lead to the development of new antibiotic-resistant pathogens [35,36].

For this reason, in this study we tested the susceptibility of *A. muciniphila* DSM 22959 to several antibiotics (chloramphenicol, ampicillin, clindamycin, tetracycline, gentamicin, streptomycin, kanamycin, and erythromycin) selected on the basis of the EFSA document regarding bacteria of human importance [22].

The results are shown in Table 1. As expected, the differences between *Lb. rhamnosus* GG and *A. muciniphila* DSM 22959 were remarkable for the majority of the tested antibiotics, since *Lb. rhamnosus* GG and *A.* *muciniphila* are Gram positive and Gram negative bacteria, respectively. 

The antibiotic susceptibility of *Lb. rhamnosus* GG was already reported in a previous study [35], and it was repeated only for comparative purposes. In detail, results confirmed the susceptibility of the probiotic strain *Lb. rhamnosus* GG to tested antibiotics, with the exception of kanamycin.

Instead, *A. muciniphila* was resistant to half of the tested antibiotics, i.e., to chloramphenicol, clindamycin, streptomycin and erythromycin. To the best of our knowledge, only Dubourg et al. [37] assessed the resistance of *A. muciniphila* to different antibiotics, but they tested antimicrobial substances different from those used in the present study. Moreover, Guo et al. [38] observed the acquisition of antibiotic resistance genes during *A. muciniphila* evolution from other species present in the human intestine, induced by the high levels of antibiotics in this environment. 

However, in the same field, Cani et al. [12] reported that the inspection of the genome sequence of *A. muciniphila* did not reveal antibiotic resistance genes that are linked to known genetically transferrable elements.

### 3.2. Hydrophobicity

The adhesion ability of bacteria plays an important role in the intestinal colonization. Therefore, this property has been considered as a potential probiotic marker along with other desirable attributes used for the selection of novel probiotic strains [39]. As it is difficult to investigate bacterial adhesion *in vivo*, an interest has been drawn in the development of *in vitro* models for preliminary screening of potentially adherent strains. For this reason, effective methods for controlling microbial adhesion have been studied [40]. In particular, the physical and chemical properties of the bacterial cell surface as the hydrophobicity and the auto-aggregation ability, could give some information on the ability of a strain to interact with its environment [41,42]. Specifically, the hydrophobicity is most likely due to complex interactions between positive and negative charges, between hydrophobic and hydrophilic components characterizing the bacterial surface [24,25,43]. 

The BATH test has been extensively used for evaluating cell surface hydrophobicity in lactic acid bacteria [24,28,43]. 

Studies on the microbial cell surface have shown that the presence of (glycol-) proteinaceous material results in higher hydrophobicity, whereas hydrophilic surfaces are associated with the presence of polysaccharides [24,29]. Hydrophobic cell surfaces were demonstrated to be highly adherent—more than 40%—to apolar solvents [24,43].

Moreover, Collado et al. [24] have found that hydrophobicity is related to auto-aggregation properties, since probiotic strains with high adhesion in the presence of hydrocarbons have shown high capability of auto-aggregation. 

Starting from these considerations, in the present study the hydrophobicity of tested strains was evaluated using xylene and toluene, and the results are reported in Table 2. 

The strains showed different hydrophobic features. In particular, *A. muciniphila* DSM 22959 showed lower levels of hydrophobicity compared to *Lb. rhamnosus* GG. Furthermore, *Lb. rhamnosus* GG showed higher levels of adhesion to xylene compared to toluene. On the other hand, *A. muciniphila* DSM 22959 showed slightly higher adhesion to toluene.

The results obtained for *A. muciniphila* DSM 22959 are not comparable with other studies, due to the lack of information. On the other hand, data regarding *Lb. rhamnosus* GG are partially confirmed by those reported in the literature. Collado et al. [24] and Tuo et al. [44] observed a hydrophobicity of *Lb. rhamnosus* GG with xylene similar to that recorded in this study (64% after 60 min), while Xu et al. [45] found values of hydrophobicity in the presence of xylene slightly lower than 50%. 

Lactobacilli could be classified into three groups: those with low (0% to 35%), moderate (36% to 70%), and high hydrophobicity (71% to 100%) [46]. Based on these ranges, and arbitrarily using them for *A. muciniphila* as well, our results indicate that *Lb. rhamnosus* GG and *A. muciniphila* DSM 22959 had a moderate hydrophobicity. 

### 3.3. Auto-Aggregation

In order to exert beneficial effects, probiotics need to achieve an adequate mass through aggregation. Consequently, the ability of probiotics to aggregate is considered a desirable property [47]. In fact, auto-aggregation ability of probiotics seems to have influence on their adhesion to intestinal epithelial cells [47,48]. The auto-aggregation ability of tested strains was determined at different times (1, 2, 5, and 24 h). In general, the auto-aggregation ability increased over time in accordance with results obtained by other authors [24,49] (Table 3).

In particular, after 24 h, *Lb. rhamnosus* GG showed a percentage of aggregation of 65.21%—very similar to that observed by Collado et al. [24]. *A. muciniphila* DSM 22959 showed not only a slightly higher percentage of aggregation at 24 h (69.42%), but also a faster auto-aggregation ability. In fact, after 2 h, it reached a percentage of 28.7% compared to that of 15.19% showed by *Lb. rhamnosus* GG. 

Tuo et al. [44] reported a significant correlation (*p* < 0.01) between auto-aggregation and hydrophobicity of some *Lactobacillus* strains. Our results confirmed the hypothesis that the BATH test is related to auto-aggregation properties only for *Lb. rhamnosus* GG, but not for *A. muciniphila.* For this strain, no correlation between hydrophobicity and auto-aggregation was observed (data not shown).

### 3.4. Biofilm Formation 

The experiments performed in this study allowed to measure the rate of adherence and subsequent biofilm formation of tested bacteria. 

*Lb. rhamnosus* GG, as reported by some authors, does not form biofilms in MRS; therefore, this medium was omitted from the experiment [50] and TSB was used [26]. 

In order to study the influence of glucose on biofilm formation, TSB without glucose or with different concentrations of this sugar was used. 

The results, summarized in Table 4, showed that *Lb. rhamnosus* GG was able to form biofilms inall conditions. In detail, it was strongly adherent (SA), i.e., capable of forming biofilms, with 0%, 0.25%, and 1.0% of glucose, while, it was moderately adherent (MA) at a concentration of 2.5% of glucose. These results showed that a high presence of glucose caused a reduction of biofilm formation in *Lb. rhamnosus* GG.

On the other hand, *A. muciniphila* DSM 22959 was classified as weakly adherent (WA) in all tested cases, regardless of the glucose concentration used. 

### 3.5. Co-Culture of A. muciniphila DSM 22959 and Lb. rhamnosus GG

Co-culture is an experimental model of rather simple culture, which allows the study of interactions between different species, likewise to what would happen *in vivo*, where the different microorganisms interact with each other, exchanging chemical signals, metabolites, etc. [51].

In this test, an *in vitro* model was set up to evaluate the interactions between *A. muciniphila* DSM 22959 and *Lb. rhamnosus* GG. The purpose of this test was to determine whether the two strains were negatively or positively influenced by each other or were not affected at all. Individual cultures of *A. muciniphila* DSM 22959 and *Lb. rhamnosus* GG were used as controls. 

On the basis of the results obtained by the antimicrobial susceptibility test, kanamycin and chloramphenicol were selected to count *A. muciniphila* DSM 22959 and *Lb. rhamnosus* GG in co-culture. In fact, *A. muciniphila* DSM 22959 showed a high sensitivity to kanamycin while *Lb. rhamnosus* GG was resistant to this antibiotic. In the same manner, *Lb. rhamnosus* GG was susceptible to chloramphenicol, while *A. muciniphila* DSM 22959 was resistant.

**Figure 1:**
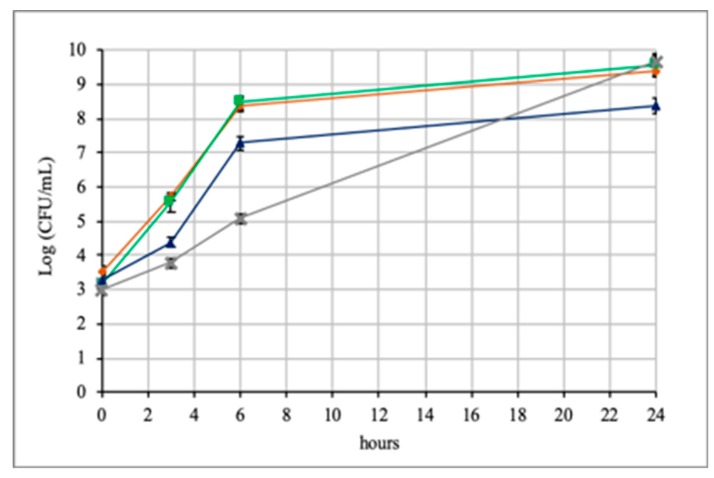
Growth of *A. muciniphila* DSM 22959 and *Lb. rhamnosus* GG in co-culture (*A. muciniphila* = 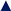; *Lb. rhamnosus* = 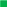; *A. muciniphila* in co-culture = **X**; *Lb. rhamnosus* in co-culture 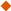).

Figure 1 shows the results obtained by the growth in co-culture. For *A. muciniphila* DSM 22959 significant differences were observed if it grew alone or in co-culture with *Lb. rhamnosus* GG. In the first hours *A. muciniphila* grew faster alone than in co-culture, and this difference was particularly evident at 6 h. Subsequently, the situation completely changed and at 24 h, *A. muciniphila* DSM 22959 alone reached 8.4 Log CFU/mL, while in co-culture it reached a value of 9.6 Log CFU/mL. Therefore, even if the growth of *A. muciniphila* in co-culture was initially slower, it was favored by the presence of *Lb. rhamnosus* GG during time. 

Contrarily to previous results, no differences were observed for *Lb. rhamnosus* GG. In fact, for this strain the value reached after 24 h in co-culture (9.5 Log CFU/mL) was very similar to that of *Lb. rhamnosus* GG cultured alone (9.6 Log CFU/mL). Therefore, *Lb. rhamnosus* GG was not influenced by the presence of *A. muciniphila* DSM 22959.

Based on these results, it is possible to state that *Lb. rhamnosus* GG positively influenced the growth of *A. muciniphila in vitro*. In fact, after 24 h *A. muciniphila* in co-culture reached a value of 1.2 Log higher than that reached alone.

The ability of *Lb. rhamnosus* GG to stimulate the growth of *A. muciniphila* DSM 22959 is an interesting result, considering that *Lb. rhamnosus* GG is widely used in probiotic preparations. However, this positive influence will have to be confirmed *in vivo*, where many other variables are involved.

### 3.6. Co-aggregation

Co-aggregation is an important feature for probiotics, because it may play a crucial role in the prevention of colonization of gastrointestinal tract (GIT) by pathogens [48]. In fact, probiotic strains can form a barrier that prevents colonization by pathogens through co-aggregation [52]. Moreover, co-aggregation with a potential pathogen allows probiotics to produce antimicrobial substances in very close proximity to them, which may inhibit the growth of pathogenic strains in the GIT [44]. 

In this study, we examined the ability of *Lb. rhamnosus* GG and *A. muciniphila* DSM 22959 to co-aggregate with some commensal/pathogen bacteria such as *E. coli*, *Proteus mirabilis*, *Enterococcus faecalis*, *Staphylococcus aureus*, and *Lb. acidophilus*. The results of this test are shown in Table 5.

In general, *Lb. rhamnosus* GG showed co-aggregation abilities with all bacteria tested, but with different percentages. After 24 h of incubation, *Lb. rhamnosus* GG showed high ability to co-aggregate with *P. mirabilis* ATCC 29906 (66.86%), *E. coli* DSM 5698 (60.40%) and *E. coli* K12-DH5 (58.30%), in agreement with Collado et al. [24]. Moreover, *Lb. rhamnosus* GG showed the highest ability to co-aggregate with *Lb. acidophilus* (72.26%).

*A. muciniphila* showed a good ability of co-aggregation with *Ec. faecalis* ATCC 2912 (44.31%), and *S. aureus* ATCC 29213 (40.48%), comparable to that observed for GG, and with *P. mirabilis* ATCC 29906 (47.46%). Moreover *A. muciniphila* also showed high co-aggregation percentages with *Lb. acidophilus* ATCC 4356.

According to other authors [46], the co-aggregation percentage was found to depend on the contact time.

Several authors suggest that the ability to co-aggregate with pathogens can be used for the preliminary selection of probiotic strains [24,52,53], considering that co-aggregation was demonstrated to be strain specific.

### 3.7. Antimicrobial Activity

Human gastrointestinal tract is colonized by a vast community of commensal microorganisms that have important effects on immune function, nutrient processing, and a broad range of other host activities. These microorganisms can influence each other positively or negatively. The interactions that are established among the components of the microbiota can strongly affect the human health. For these reasons, it is important to know the interaction that occur between probiotics and commensal and/or pathogen microorganisms. In this field, we examined the interactions between *Lb. rhamnosus* GG, *A. muciniphila* DSM 22959, and the same commensal/pathogen bacteria used for the co-aggregation assay. The agar well diffusion technique was used to detect if the growing cells of *Lb. rhamnosus* GG and *A. muciniphila* DSM 22959 (producers), had inhibitory properties against the indicator strains.

For *A. muciniphila* DSM 22959 no halos of inhibition were found, evidencing that it was unable to inhibit all indicators (data not shown). Similar results were observed for *Lb. rhamnosus* GG, which exerted a moderate antimicrobial activity only against *E. coli* DSM5698 (diameter of inhibition halo 2.4 mm).

In conclusion, to the best of our knowledge, this is the first study that evaluated *A. muciniphila* for cell surface properties and some other features considered important for the screening of probiotic bacteria. For some characteristics, such as auto-aggregation, co-aggregation, hydrophobicity (toluene), and antimicrobial activity, the results obtained for *A. muciniphila* DSM 22959 were comparable with those of *Lb. rhamnosus GG*.

The results related to sensitivity to antibiotics revealed a higher resistance of *A. muciniphila* DSM 22959 then that showed by *Lb. rhamnosus* GG. This latter aspect certainly deserves a specific study, with particular attention to the presence of potentially transferable resistance genes.

Furthermore, interesting results derived from the positive effect of *Lb. rhamnosus* GG on the growth of *A. muciniphila* when co-cultured.

Regarding the possible future use of *A. muciniphila* as a probiotic, it should be pointed out that there are no published human clinical trials that have studied the effects of *A. muciniphila* intake in humans, leading to a lack of conclusive evidence on its safety, and also the results obtained in this study are not conclusive on this point.

However, we agree with Brodmann et al. [54], who raise the question of whether it is necessary to prove the safety of an organism that is already part of nutrition in the early stages of life [14] and which constitutes 3% to 5% of the gut microbiota [1,2].

Further insights are needed on this topic. The guidelines on probiotics as well as the European legislation should take specific cases such as *A. muciniphila* into account, as suggested by Brodmann et al. [54].

## Figures and Tables

**Table 1:** Antimicrobial susceptibility of *A. muciniphila* DSM 22959 and *Lb. rhamnosus* GG against chloramphenicol (CHL), ampicillin (AMP), clindamycin (CLI), tetracycline (TET), gentamicin (GEN), streptomycin (STR), kanamycin (KAN), and erythromycin (ERY), as determined with Etest. MIC values were expressed as µg/mL.

Strains	CHL	AMP	CLI	TET	GEN	STR	KAN	ERY
***A. muciniphila* DSM 22959**	256	2	256	0.75	4	128	12	64
***Lb. rhamnosus* GG**	3	1.5	0.5	0.5	8	24	256	0.064
Cut-off values [22]	**4**	**4**	**1**	**8**	**16**	**32**	**64**	**1**

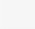
 Resistant; 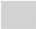 Susceptible.

**Table 2:** Adhesion of *A. muciniphila* DSM 22959 and *Lb. rhamnosus* GG to hydrocarbons (expressed as hydrophobicity %) measured using the BATH test after 15, 30, and 60 min (contact time).

Strains	Contact Time (min)	Xylene	Toluene
***A. muciniphila* DSM 22959**	15	6.9 (±0.5)	25.6 (±0.4)
	30	38.0 (±0.6)	40.7 (±0.6)
	60	39.4 (± 0.3)	43.7 (± 0.4)
***Lb. rhamnosus* GG**	15	55.9 (±0.3)	48.2 (±0.4)
	30	64.0 (±0.6)	48.5 (±0.3)
	60	64.0 (±0.4)	49.4 (±0.6)

**Table 3:** Auto-aggregation percentages of *A. muciniphila* DSM 22959 and *Lb. rhamnosus* GG strains evaluated after 1, 2, 5, and 24 h of incubation.

	% Auto-Aggregation			
Strains	1 h	2 h	5 h	24 h
***A. muciniphila* DSM 22959**	3.59 ± 0.29	28.70 ± 1.38	30.94 ± 2.36	69.42 ± 4.51
***Lb. rhamnosus* GG**	4.67 ± 0.12	15.19 ± 0.73	20.13 ± 1.0	65.21 ± 2.9

**Table 4:** Biofilm formation of *A. muciniphila* DSM 22959 and *Lb. rhamnosus* GG after incubation at 37 °C for 24 h in TSB containing different concentrations of glucose (0%, 0.25%, 1%, and 2.5%). The cut-off (OD_C_) was defined as the mean OD value of the negative control. Based on the OD, strains were classified as not adherent (OD ≤ OD_C_), weakly adherent (OD_C_ ˂ OD ≤ 2× OD_C_), moderately adherent (2× OD_C_ ˂ OD ≤ 4× OD_C_) or strongly adherent (4× OD_C_ ˂ OD).

Strains	TSB 0%	TSB 0.25%	TSB 1%	TSB 2.5%
*A. muciniphila* DSM 22959	**WA**	**WA**	**WA**	**WA**
*Lb. rhamnosus* GG	**SA**	**SA**	**SA**	**MA**

SA = strongly adherent; MA = moderately adherent; WA = weakly adherent.

**Table 5:** Co-aggregation percentages of *Lb. rhamnosus* GG and *A. muciniphila* DSM 22959 with *E. coli* DSM 5698, *E. coli* K12-DH5, *P. mirabilis* ATCC 29906, *Ec. faecalis* ATCC 2912, *S. aureus* ATCC 29213, and *Lb. acidophilus* ATCC 4356 evaluated after 5 and 24 h of incubation.

	*Co-Aggregation*			
Strains	*Lb. rhamnosus* GG		*A. muciniphila* DSM 22959	
	5 h	24 h	5 h	24 h
***Ec. faecalis* ATCC2912**	16.39 ± 1.8	40.64 ± 3.6	23.81 ± 2.2	44.31 ± 4.0
***P. mirabilis* ATCC 29906**	26.20 ± 1.9	66.86 ± 3.1	24.51 ± 2.3	47.46 ± 3.9
***S. aureus* ATCC 29213**	8.29 ± 0.7	39.23 ± 1.3	20.64 ± 3.3	40.48 ± 3.6
***E. coli* DSM 5698**	35.70 ± 8.1	60.40 ± 2.9	2.67 ± 0.11	6.15 ± 0.25
***E. coli* K12-DH5**	34.80 ± 3.5	58.30 ± 2.25	2.31 ± 0.21	6.10 ± 0.30
***Lb. acidophilus* ATCC 4356**	40.06 ± 3.5	72.26 ± 6.1	24.77 ± 3.6	54.38 ± 4.2
